# Long-term follow-up of patients with phenylketonuria treated with tetrahydrobiopterin: a seven years experience

**DOI:** 10.1186/s13023-015-0227-8

**Published:** 2015-02-08

**Authors:** Iris Scala, Daniela Concolino, Roberto Della Casa, Anna Nastasi, Carla Ungaro, Serena Paladino, Brunella Capaldo, Margherita Ruoppolo, Aurora Daniele, Giuseppe Bonapace, Pietro Strisciuglio, Giancarlo Parenti, Generoso Andria

**Affiliations:** Department of Translational Medicine-Section of Pediatrics, Federico II University, Via S. Pansini 5, 80131 Naples, Italy; Department of Pediatrics, Magna Graecia University, Catanzaro, Italy; Department of Clinical Medicine and Surgery, Physiology Nutrition Unit, Federico II University, Naples, Italy; Department of Clinical Medicine and Surgery, Federico II University, Naples, Italy; Department of Molecular Medicine and Medical Biotechnology, Federico II University, Naples, Italy; CEINGE-Biotecnologie Avanzate s.c.ar.l., Naples, Italy; Dipartimento di Scienze e Tecnologie Ambientali Biologiche Farmaceutiche, Seconda Università degli Studi di Napoli, Caserta, Italy

**Keywords:** Phenylalanine hydroxylase deficiency, Phenylketonuria, Tetrahydrobiopterin, Sapropterin, Hyperphenylalaninemia, Therapy, Tolerance, Safety, Side effects

## Abstract

**Background:**

Phenylketonuria (PKU) is an autosomal recessive disorder caused by the deficiency of phenylalanine hydroxylase that catalyzes the conversion of phenylalanine to tyrosine, using tetrahydrobiopterin (BH4) as coenzyme. Besides dietary phenylalanine restriction, new therapeutic options are emerging, such as the treatment with BH4 in subgroups of PKU patients responding to a loading test with BH4.

**Methods:**

A no-profit open-label interventional trial with long-term oral BH4 therapy, sponsored by the Italian Medicines Agency (AIFA), was performed in a group of 17 PKU patients resulted as BH4 responders among 46 subjects analyzed for BH4-responsiveness (prot. FARM5MATC7). We report on efficacy and safety data of BH4 therapy and analyze factors predicting BH4-responsiveness and long-term response to BH4. A BH4-withdrawal test was used as a proof of the efficacy of long-term therapy with BH4.

**Results:**

Forty-four percent of the patients responded to the 48 h-long loading test with BH4. All the phenotypic classes were represented. Genotype was the best predictor of responsiveness, along with lower phenylalanine levels at diagnosis, higher tolerance and lower phenylalanine/tyrosine ratio before the test. In BH4 responder patients, long-term BH4 therapy resulted safe and effective in increasing tolerance while maintaining a good metabolic control. The BH4 withdrawal test, performed in a subset of patients, showed that improved tolerance was directly dependent on BH4 assumption. Tolerance to phenylalanine was re-evaluated in 43.5% of patients and was longitudinally analyzed in 5 patients.

**Conclusions:**

Long-term treatment with BH4 is safe and effective in increasing tolerance to phenylalanine. There is real need to assess the actual tolerance to phenylalanine in PKU patients to ameliorate quality of life, improve nutritional status, avoiding unnecessarily restricted diets, and interpret the effects of new therapies for PKU.

**Electronic supplementary material:**

The online version of this article (doi:10.1186/s13023-015-0227-8) contains supplementary material, which is available to authorized users.

## Background

Phenylketonuria (PKU; MIM 261600) is an autosomal recessive disorder caused by the deficiency of phenylalanine hydroxylase (PAH, EC 1.14.16.1), a liver enzyme that catalyzes the conversion of phenylalanine (Phe) to tyrosine (Tyr), using tetrahydrobiopterin (BH4) as coenzyme. To date, more than 500 causative mutations of the PAH gene have been identified (http://www.pahdb.mcgill.ca; http://www.biopku.org). In spite of the continuum spectrum of phenotypes, PKU is classified into classic PKU (cPKU), moderate PKU (moPKU) and mild PKU (mPKU) according to plasma Phe levels at diagnosis and tolerance, defined as the highest Phe intake able to keep blood Phe levels within the safe range [1]. In addition, hyperphenylalaninemia is divided in mild hyperphenylalaninemia (HPA)-gray zone, requiring dietary protein restriction, and mild hyperphenylalaninemia when Phe levels are below 360 micromol/L and no treatment is required [1]. Untreated PKU leads to neurological abnormalities, while early treatment with a Phe-restricted diet prevents brain damage and results in almost normal neurological development [2]. Unfortunately, this dietary regimen is often complicated by psychological discomfort and reduced compliance to the diet after the first years of life. Initial reports supported the possibility to loosen diet during adulthood; however, as defects of executive functions and brain hypomyelination may occur in patients with poor metabolic control [3,4], a life-long restricted dietary therapy has been recommended. Since 1999, several independent reports have shown that BH4 can decrease plasma Phe levels and increase Phe oxidation to Tyr [5-19] by acting as a chemical chaperone [20,21] in patients defined as BH4 responders. Responsiveness to BH4 has been arbitrarily defined as a reduction of at least 30% of plasma Phe levels during a loading test with BH4 compared to the baseline Phe value [22]. The prevalence of BH4 responsiveness is variable in different studies, ranging from 20% [9] to 62% [23]. Besides the interplay among the PAH enzyme, the coenzyme (BH4) and the substrate (Phe), the difference in the rate of BH4-responder patients also resides in the protocols varying in duration (24 h, 48 h, 1 week or 4 weeks) and in BH4 testing dose (10 mg/kg vs 20 mg/kg of body weight) [24]. Reports on long-term treatment with BH4 show that BH4 allows a better metabolic control and a diet relaxation without serious adverse effects. Two randomized placebo-controlled trials reported a significant lowering of blood Phe concentration in the BH4 group after 6 weeks [11] and improvement of Phe tolerance after 10 weeks [13]. The improvement of Phe intake with BH4 was also observed in subsequent studies [13,15-19,25]. Recent reports attempted to establish predictors of BH4 responsiveness and of BH4 long-term response, including Phe levels at diagnosis, Phe/Tyr ratio, Phe tolerance before BH4 treatment and genotype [19]. US recommendations for the use of BH4 in PKU have been recently published, but some questions are still open such as i) variability of response to BH4 and therapeutic effects in subpopulations, ii) long-term efficacy and safety outcomes, iii) effects of diet liberalization on nutritional status and iv) risk of dietary overtreatment, especially in critical periods such as infancy, adolescence or pregnancy [1].

In this study, we show the results of a no-profit open-label interventional trial with long-term oral BH4 therapy. The trial started in 2006 and ended in September 2009, date of marketing of sapropterin dihydrochloride (Kuvan®, Merck-Serono), the synthetic form of BH4. A long-term follow-up continued from September 2009 to December 2013. We report on efficacy and safety data of BH4 therapy and analyze factors predicting BH4-responsiveness and long-term response to BH4. We finally report on a BH4-withdrawal test, used as a proof of the efficacy of long-term therapy with BH4, and on the reassessment of Phe tolerance in a subgroup of patients who underwent the BH4 responsiveness protocol.

## Methods

### Patients

Forty-six patients with HPA due to PAH deficiency were recruited at the Federico II University, Naples, and the Magna Graecia University, Catanzaro, Italy after ethical committee approval (Prot. n. 239/06) and written informed consent.

Inclusion criteria were: age >4 years, PKU/hyperphenylalaninemia requiring dietary restriction, complete genotyping of PAH gene, compliance to study procedures, provision of a written informed consent. Patients with concurrent diseases that would interfere with enrolment or safety, with concomitant drug treatment, with clinical diagnosis of primary BH4 deficiency or unavailable for study participation were excluded from the study. Patients fulfilling the inclusion criteria entered into the study protocol supported by the Italian Medicines Agency (AIFA) (prot. FARM5MATC7; EudraCT code: 2006-005768-22). Patients were phenotypically classified according to Phe blood value before dietary treatment and to Phe tolerance, determined at 5 years of age (historical tolerance). Four different phenotypes were identified: 1) classic PKU (cPKU; Phe >1200 micromol/L; tolerance <350 mg/day), 2) moderate PKU (moPKU; Phe 900-1200 micromol/L; tolerance 350-400 mg/day), 3) mild PKU (mPKU; Phe 600-900 micromol/L; tolerance 400-600 mg/day), and 4) mild HPA-gray zone (henceforth called mHPA; 360-600 micromol/L; tolerance >600 mg/day), requiring dietary protein restriction [1]. When a discrepancy was noted between pre-treatment blood Phe levels and Phe tolerance, patients were classified according to Phe tolerance. Thirty-nine percent of patients were cPKU, 14% were moPKU, 35% were mPKU and 12% were mHPA requiring low protein diet. Age at loading test, gender, phenotypic class, molecular analysis and predicted residual activity (PRA) of each mutation in responders and non-responders, as reported in the BIOPKU database (http://www.biopku.org), are shown in Additional file 1. All patients were genotyped by direct sequencing of the PAH gene [26]. Plasma Phe and Tyr were analyzed by amino acid analyzer (Beckman System 6300).

### Assessment of BH4 responsiveness

#### Pre-loading test protocol

Each patient was asked to comply to the assigned diet with Phe amounts corresponding to the known Phe tolerance and equally distributed in three main meals. Patients were asked to fill a meal diary, reviewed by the dietician. The dietary regimen had to be followed for at least two weeks before the BH4 loading test. In the week before the BH4 loading test, blood Phe and Tyr were analyzed twice a week after an overnight fast to verify the compliance to diet on the basis of Phe values. Only a 15% variance between the two Phe values was accepted. If plasma Phe resulted ≥ 400 micromol/L, the BH4 loading test was started; if plasma Phe resulted < 400 micromol/L, dietary Phe intake was increased every 10-15 days until blood Phe resulted ≥ 400 micromol/L.

#### BH4 loading test

A 48 h-long BH4 loading test was performed with BH4 tablets (Schircks Laboratories, Switzerland) in two oral doses of 20 mg/kg at T0 and T24 hours. Plasma Phe and Tyr were analyzed at 0, 4, 8, 12, 24, 32 and 48 hours**.** Patients were hospitalized during the loading test to ensure optimal compliance. BH4-responsiveness was defined by a reduction of Phe levels >30% from the baseline Phe value. Patients were defined as rapid responders if >30% reduction of blood Phe levels occurred within the first 24 h of the test, and as slow-responders if the response was observed between T24 and T48.

### Long-term therapy with BH4

BH4-responder patients started a long-term treatment with BH4 at the initial dose of 10 mg/kg/day. In some patients, the dose was changed based on the metabolic control. During the trial, all patients assumed 50 mg BH4 tablets (Schircks Laboratories), divided in three oral doses before meals, until September 2009, when therapy was shifted to sapropterin. Phe intake was increased by standard food exchange lists ranging from 75 to 225 mg/day, every 15-30 days if Phe levels resulted within the target. Plasma Phe was always dosed after the overnight fast. The reference Phe values in different age groups were those reported for our country in Blau et al. [27] and in the recommendations of the Italian Society for the Study of Inborn Errors of Metabolism and Neonatal Screening [28], i.e. 120-360 micromol/L until 12 years of age and 120-600 micromol/L for older ages.

Outcome measures were improvement of metabolic control and of tolerance to Phe. Median plasma Phe obtained during the 5 years preceding BH4 treatment was used as index of metabolic control before BH4 and compared with plasma Phe levels obtained during BH4 therapy. Dietary Phe intake (mg/day) was assessed before starting and during BH4 therapy. A three days meal diary was analyzed at each visit. Response to long-term therapy with BH4 was defined by a significant increase of Phe intake compared to the historical tolerance and to tolerance before BH4.

### BH4 withdrawal test

Seven patients (2 moPKU, 3 mPKU and 2 mHPA) aged 16 to 20 years on long-term treatment (28-35 months) with sapropterin undertook a BH4 withdrawal test as a confirmatory proof of efficacy of BH4 therapy, after parents/patients’ agreement. Patients were asked to check plasma Phe levels twice during the 10 days before the test (day -10 and -5; BH4-1). BH4 therapy was then withdrawn for additional 10 days and Phe levels were assessed at day +5 and +10 from the discontinuation of the drug (Withdrawal). Patients were finally asked to re-start BH4 and to control plasma Phe levels 10 days later (day +20) (BH4-2).

### Safety and tolerability

Anamnestic records were used to investigate the occurrence of adverse events. Complete physical examination was performed monthly by the primary care physician and every three months by the physicians in each recruiting centre. Nutritional status (hemoglobin, iron, total proteins, albumin, plasma amino acids profile, vitamin B12, folic acid), liver and kidney functions were also analyzed.

### Statistical analysis

Normality of continuous data was assessed by the Levene’s test. The Student’s t-test was used to compare means and the Mann-Whitney U-test to compare medians. Related continuous data were analysed by the Wilcoxon test. Pearson's correlation coefficient (r) was used to measure the strength of the association between two variables. The Chi-square test was used for categorical data. Variance was calculated as a measure of Phe variability before and during BH4. A two-tailed p-value <0.05 was assumed as statistical significant. Statistical analysis was performed by SPSS 17.0 software.

## Results

### Reassessment of tolerance to phenylalanine

To achieve stable Phe values at the minimal concentration of 400 micromol/L, a pre-test protocol was developed. During this period, in a subgroup of patients, we noted that, despite the gradual increase of dietary Phe, plasma Phe did not increase as expected and diet adjustments had to be repeated for 2-3 times until the 400 micromol/L threshold was reached. These observations were made in 20 out of 46 enrolled patients (43.5%). Tables 1 and 2 report genotype, historical tolerance, the median plasma Phe level before enrolment and the new Phe intake. In the first set of 10 patients (Table 1), plasma Phe persistently remained <360 micromol/L despite dietary Phe increased from 1.3-to 3.2 folds compared to the baseline tolerance. A final substantial increase of dietary Phe had to be done to exceed the 400 micromol/L threshold required for the loading test. In a second group of 10 patients (Table 2), up to three diet adjustments were sufficient to reach plasma Phe values >400 micromol/L. In this group, we noted that the maximum plasma Phe concentration was 600 micromol/L, within the upper reference range for age group, and that in some subjects Phe values were not different from the median plasma Phe of the five years before the inclusion in the study. However, dietary Phe increased from 1.6- to 6.3-folds from the baseline. Neither the phenotypic class nor the genotype appeared to be predictive of the increase of Phe intake.

**Table 1:** **Patients with Phe < 360 micromol/L during the pre-loading test protocol**

**Subject (#)**	**PAH genotype**	**Historical tolerance** *	**Median plasma Phe**	**New Phe intake**
			***(10 th -90 th centile)*** ^***§***^	**[Phe values]**
3	R261Q/R158Q	400	360 (180-570)	786 [390; 376]
7	L48S/R158Q	505	246 (120-318)	1321 [264; 282]
10	R261Q/R261Q	410	204 (130-372)	817 [330; 380]
12	R261Q/P281L	410	100 (60-180)	1328 [198; 228]
13	L48S/R158Q	410	840 (300-1080)	1133 [290; 324]
14	L48S/R158Q	440	426 (200-540)	978 [300; 348]
18	L48S/R158Q	650	246 (120-360)	1342 [210; 234]
22	R261Q/IVS10nt-11G > A	345	372 (100-700)	950 [330; 360]
23	R261Q/IVS10nt-11G > A	340	336 (102-588)	660 [336; 348]
42	IVS06nt-2delA/P281L	630	336 (110-480)	850 [290; 340]

Notes. *Historical tolerance (mg Phe/day) calculated at 5 years of age; ^§^Median plasma Phe (micromol/L) calculated from Phe values of the 5 years preceding the inclusion into the study; the new Phe intake (mg Phe/day) represents the dietary Phe assigned during the pre-loading test protocol with the corresponding plasma Phe values (two determinations), reported in square brackets. In all these patients, an additional final increase of dietary Phe was made to reach Phe values ≥ 400 micromol/L as required for the BH4 loading test.

**Table 2:** **Patients with Phe >400 micromol/L during the pre-loading test protocol**

**Subject (#)**	**PAH Genotype**	**Historical tolerance** *	**Median plasma Phe**	**New Phe intake**
			***(10 th -90 th centile)*** ^***§***^	**[Phe values]**
4	R261Q/L48S	360	780 (370-900)	1335 [432; 456]
5	R261Q/P281L	395	168 (30-582)	1228 [510; 546]
6	L48S/Q301P	385	150 (100-174)	711 [492; 552]
8	L48S /D222G	450	174 (140-222)	1995 [438; 444]
9	165delT/P366H	550	510 (330-570)	3187 [590; 526]
17	R158Q/D338Y	1500	534 (360-540)	2658 [516; 486]
19	165delT/P366H	1920	540 (420-600)	3187 [516; 600]
25	R158Q/R176X	320	456 (180-864)	963 [468; 486]
39	P281L/R158Q	390	600 (318-720)	1164 [600; 582]
40	IVS10nt-11G > A/F39del(116118delTCT)	390	366 (100-444)	670 [360; 408]

Notes. *Historical tolerance (mg Phe/day) calculated at 5 years of age; ^§^Median plasma Phe (micromol/L) calculated from Phe values of the 5 years preceding the inclusion into the study; the new Phe intake (mg Phe/day) represents the dietary Phe assigned during the pre-loading test protocol with the corresponding plasma Phe values (two determinations), reported in square brackets.

### BH4 loading test

#### Subjects

Forty-three patients out of 46 correctly completed the BH4 loading test (Additional file 2). Nineteen out of 43 patients (44%) resulted as BH4-responders: 2 (10.5%) were cPKU, 4 (21%) were moPKU, 10 (52.5%) were mPKU and 3 (16%) were mHPA. Among non-responders, 19 (79%) were cPKU, 2 (8.5%) were moPKU, 1 (4%) was mPKU and 2 (8.5%) were mHPA. According to the phenotypic class, response to BH4 was detected in 9.5% of 21 cPKU, 66.6% of 6 moPKU, 91% of 11 mPKU and 60% of 5 mHPA.

#### Genotypes

The most common PAH allelic variants found among BH4-responders were p.R261Q (23.7%), p.L48S (23.7%), p.R158Q (18.4%) and p.P281L (10.5%). The latter mutation, with low predicted enzymatic activity, was associated with the p.R261Q mutation in all cases. In the BH4-responder group, all mutations were missense except two (165delT and IVS10nt-11G > A). Two patients carried the p.P366H mutation whose residual enzymatic activity is still unknown. However, as in both cases this mutation was associated with the c.165delT variant, known to be non-responsive to BH4 [29], the p.P366H mutation can be associated with BH4-responsiveness. Among BH4 non-responders, the IVS10nt-11G > A allele accounted for 19% of total alleles, followed by the p.R261Q (10.6%), the p.P281L (10.6%) and the p.R158Q (8.5%) mutations. Overall, splice mutations accounted for 42% of the total alleles found in non-responder patients.

#### Rate of response

**Figure 1:**
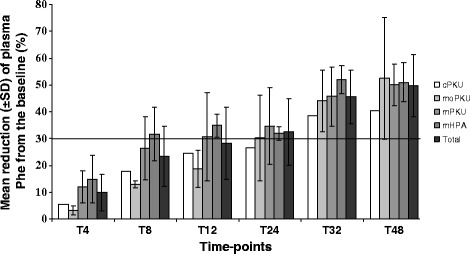
**Results of the 48 h-BH4 loading test (20 mg/kg/day) of the 19 HPA responder subjects according to their phenotypic class.** Cumulative data at each time-point are shown as mean ± SD, except for the 2 cPKU patients where only mean value is represented.

As shown in Table 3 and Figure 1, the reduction of blood Phe from the baseline among BH4 responders ranged between 33.3% and 77.1%. The rate of Phe reduction during the test was largely independent from the phenotypic class. Nine patients (47%) showed a rapid response and 10 patients (53%) a slow response. Both cPKU patients and 3 out of 4 moPKU showed a slow response; considering together mPKU and mHPA subjects, 61% showed a rapid response and 39% a slow response. Data analysis showed a tendency toward significance of the association between pre-treatment Phe at diagnosis and the rapidity of response, being lower Phe levels better predictors of a response to BH4 within the first 24 h of the test [Phe at diagnosis (mean ± SD) in rapid responders 853 ± 450 micromol/L; Phe at diagnosis (mean ± SD) in slow responders 1305 ± 500 micromol/L; p = 0.057].

**Table 3:** **Response to BH4-loading test according to the phenotypic class**

**Subject (#)**	**Phenotype**	**Genotype**	**Age at loading test (years)**	**Maximal Phe reduction from T0 (%)**	**Rapid (<24 h)/ slow response**	**Phe (T0) (** ***micromol/L)***	**Tyr (T0) (** ***micromol/L)***	**Phe/Tyr (T0)**
1	cPKU	R261Q/P281L	13	40	Slow	1052	46	22.8
2	cPKU	R261Q/P281L	11	41	Slow	644	51	12.6
3	moPKU	R261Q/R158Q	17	33.3	Slow	768	62	12.3
4	moPKU	R261Q/L48S	14	66.6	Rapid	434	44	9.8
5	moPKU	R261Q/P281L	14	36.8	Slow	689	66	10.4
6	moPKU	L48S/Q301P	7	77.1	Slow	556	44	12.6
7	mPKU	L48S/R158Q	14	48	Slow	538	59	9.1
8	mPKU	L48S/D222G	12	58	Rapid	446	67	6.6
9	mPKU	165delT/P366H	18	67	Rapid	605	42	14.4
10	mPKU	R261Q/R261Q	13	44	Rapid	680	51	13.3
11	mPKU	R261Q/IVS10nt-11G > A	20	34	Slow	600	53	11.3
12	mPKU	R261Q/P281L	17	47.3	Slow	1024	45	22.7
13	mPKU	L48S/R158Q	21	60	Rapid	726	53	13.6
14	mPKU	L48S/R158Q	10	53	Slow	471	61	7.7
15	mPKU	L48S/L48S	5	80	Rapid	690	48	14.3
16	mPKU	R158Q/Y414C	12	52	Rapid	665	50	13.3
17	mHPA	R158Q/D338Y	14	54.6	Rapid	519	54	9.6
18	mHPA	L48S/R158Q	19	45	Slow	522	54	9.6
19	mHPA	165delT/P366H	22	59	Rapid	566	38	14.8

The percentage of Phe reduction during the loading test was greater among rapid responders compared to slow-responders [mean % of reduction ± SD in rapid responders: 60 ± 10; mean % of reduction ± SD in slow responders: 45.5 ± 12; p = 0.009] and was inversely correlated with Phe values during the 5 years prior to the BH4 loading test (r = -0.6; p = 0.008). The percentage of Phe reduction during the test correlated neither with Phe at diagnosis nor with tolerance (p > 0.05) but was associated with the genotype and was greater when two alleles with PRA > 1% were present (p < 0.05). Taken together these observations indicate that all phenotypic classes are eligible for the assessment of response to BH4 and that all patients should be advised to perform a 48 h-long loading test, even if the majority of milder HPA phenotypes will respond within the first 24 h of the test.

#### Predictors of BH4 responsiveness

Lower Phe levels at diagnosis, higher Phe tolerance, the presence of at least one allele with residual enzymatic activity and lower Phe/Tyr ratio at T0 were predictors of response to BH4 (Table 4).

**Table 4:** **Predictors of BH4-responsiveness**

	**BH4 responders (N = 19)**	**BH4 non-responders (N = 24)**	**p-value**
Gender	11 M/8 F	9 M/15 F	0.18
Age (*years*) at BH4 loading test *(Mean ± SD)*	14.4 ± 4.5	13.6 ± 4.8	0.63
Phe^§^ at diagnosis *(Mean ± SD)*	1148 ± 613	1612 ± 816	**0.04**
Phe tolerance* *[Mean (range)]*	549 (235-1920)	335 (220-630)	**0.02**
Genotype One allele with PRA > 1%	17	14	**0.02**
Two alleles with PRA > 1%	13	5	**0.001**
Phe^§^ at loading test (T0) *(Mean ± SD)*	615 ± 180	695 ± 228	0.2
Tyr^§^ at loading test (T0) *(Mean ± SD)*	52.35 ± 8.5	48.12 ± 12.4	0.36
Phe/Tyr at loading test (T0)	12.58 ± 4.5	16.9 ± 5.1	**0.01**

*Phe tolerance (mg/day), as calculated at 5 years of age. ^§^ Phe and Tyr plasma concentrations are expressed in micromol/L. PRA: predicted residual activity.

### Long-term therapy with BH4

Two BH4-responder patients (#15, 16) did not agree to the long-term treatment with BH4. Seventeen patients (2 cPKU, 4 moPKU, 8 mPKU and 3 mHPA) started a long-term treatment with BH4 (Schircks Laboratories). Five patients were adults when BH4 therapy was started (#9, 11, 13, 18, 19). From 2009, all patients shifted to sapropterin (Kuvan®) and all started with one morning dose, as indicated by the manufacturer. However, over a short-term follow-up, 15/17 patients (88%) worsened their metabolic control. The daily dose was again divided in three and the Phe values returned to the previous levels. The follow-up ranged from 60 to 84 months, with the exception of patient #11 who resulted as pseudo-responder and discontinued the therapy after 12 months. At the end of the follow-up period, 12 patients were adults (age range 18-29 years). Patients who resulted as BH4 responders and started the long-term treatment during adolescence, maintained the same metabolic control at the same dietary regimen during adulthood.

Tolerance increased in all cases by 2.3- to 11.6-folds compared to the historical tolerance, as calculated at 5 years of age (Table 5). Mean tolerance was 583 ± 443 mg Phe/day before BH4 therapy and 2798 ± 1568 mg Phe/day during BH4 treatment (p < 0.0001). Significant increases of Phe intake were also observed when tolerance on BH4 therapy was compared to the reassessed Phe intake before BH4, as reported in Tables 1 and 2. Nine out of 17 patients (53%) reached a Phe intake ≥ 3000 mg/day with no need of amino acids and vitamin supplements. Two mPKU patients (#10, 12) reached a daily Phe intake of 2392 and 2278 mg/day, respectively and needed only small amounts of amino acids and vitamin supplements.

**Table 5:** **Phe tolerance and plasma Phe of responder patients on long-term treatment with BH4**

**Subject (#)**	**Phenotype**	**Follow-up (months)**	**Tolerance at 5 years of age**	**Tolerance before BH4 therapy**	**Tolerance on BH4 therapy**	**Median plasma Phe pre-BH4** ^**§**^ **(10 th -90 th centile)**	**Median plasma Phe on BH4 (10 th -90 th centile)**
**1**	cPKU	80	280	280	1180	552 (120-588)	492 (276-600) ^NS^
**2**	cPKU	60	325	325	1010	240 (180-300)	480 (246-522)*
*Mean*			302.5	302.5	1095		
*Median (10 th -90 th centile)*						426 (210-780)	492 (300-600) ^NS^
**3**	moPKU	84	400	786	1386	360 (180-570)	468 (384-600) ^NS^
**4**	moPKU	80	360	1335	4185	780 (370-900)	140 (102-170)*
**5**	moPKU	79	395	1228	4000	366 (100-444)	312 (180-354)*
**6**	moPKU	66	385	711	1611	150 (100-174)	258 (90-282)**
*Mean ± SD*			385 ± 17*	1015 ± 312*	2795.5 ± 1502		
*Median (10 th -90 th centile)*						310 (90-756)	495 (180-780)**
**7**	mPKU	80	505	1321	3800	246 (120-318)	540 (240-660)**
**8**	mPKU	75	450	1995	4700	174 (140-222)	228 (126-360)^NS^
**9**	mPKU	75	550	3187	5000	510 (330-570)	560 (372-600)^NS^
**10**	mPKU	60	410	817	2392	204 (130-378)	450 (348-600)**
**11**	mPKU	12	440	440	1188	162 (100-300)	600 (400-660)**
**12**	mPKU	60	410	1328	2278	100 (60-180)	456 (360-600)**
**13**	mPKU	61	410	1133	3000	840 (300-1080)	516 (264-600)^NS^
**14**	mPKU	61	440	978	1950	426 (200-540)	402 (216-540)^NS^
*Mean ± SD*			451 ± 50.7**	1400 ± 851*	3038 ± 1351		
*Median (10 th -90 th centile)*						240 (90-660)	534 (200-780)**
**17**	mHPA	84	1500	2658	5000	534 (360-540)	250 (170-300)**
**18**	mHPA	80	650	1342	4000	246 (120-360)	480 (360-600)^NS^
**19**	mHPA	72	1920	3187	4500	540 (420-600)	360 (270-600)*
*Mean ± SD*			1356 ± 637**	2395 ± 950*	4500 ± 500		
*Median (10 th -90 th centile)*						468 (204-570)	432 (210-600)^NS^
***Overall*** *(Mean ± SD)*			583 ± 443**	1627 ± 988**	2798 ± 1568		
***Overall*** *Median (10 th -90 th centile)*						320 (130-730)	490 (198-770)**

^**§**^Calculated from Phe concentrations (micromol/L) of the 5 years preceding the start of BH4 therapy; Wilcoxon Z-test was used for intrasubject analysis; Mann-Whitney U-test was used to compare patients’ groups. *p < 0.05; **p < 0.01, NS: not significant. Tolerance (mg Phe/day) on BH4 was compared to both tolerance calculated at 5 years of age and tolerance before BH4 therapy for patients # 3, 4, 5, 6, 7, 8, 9, 10, 12, 13, 14, 17, 18, 19 (see Tables 1 and 2). For patients # 1, 2 and 11, tolerance pre-BH4 was consistent with tolerance at 5 years of age. For the cPKU group (only two patients), no statistical analysis is given for tolerance.

In spite of the increase of dietary Phe, both groups of patients treated either with sapropterin plus diet or with sapropterin alone maintained an optimal metabolic control. Six patients out of 17 (23.5%) showed median blood Phe ≤360 micromol/L; the remaining subjects had median Phe values <600 micromol/L, within the recommended range for age group considered for the study (Table 5). Intra-subject analysis showed that median plasma Phe levels on BH4 significantly decreased in 4 patients, remained stable in 7 patients and increased in 6 patients compared to the median plasma Phe pre-BH4 (Table 5). When all patients data were combined, median plasma Phe levels on BH4 raised from 320 to 490 micromol/L, although remaining into the reference range for age group (Table 5). Analysis of variance of Phe levels before and during BH4 failed to show a significant decrease of Phe variability on BH4 therapy (p = 0.6).

The new tolerance under BH4 therapy significantly correlated with the historical tolerance (r = 0.52; p = 0.03). The percentage of Phe reduction during the loading test and the rapid *vs* slow response to BH4 did not correlate with the new tolerance and the median Phe values under BH4.

No difference was noted in nutritional status under BH4 therapy. During the treatment, body mass index (BMI) of all BH4 responders increased from 21,7 ± 2,1 to 23,6 ± 1,39 (p = 0.006). This difference was detected among patients treated with BH4 + diet and was mainly present in mPKU and moPKU patients (p = 0.008 and p = 0.02, respectively; Additional file 3).

All patients were confirmed as true responders to BH4 after a long-term follow-up, with the exception of patient #11. Notably, other 3 patients with the same genotype resulted non-responder to BH4, thus suggesting that the genotype p.R261Q/IVS10nt-11G > A is predictive of non-responsiveness.

### BH4 withdrawal test

**Figure 2:**
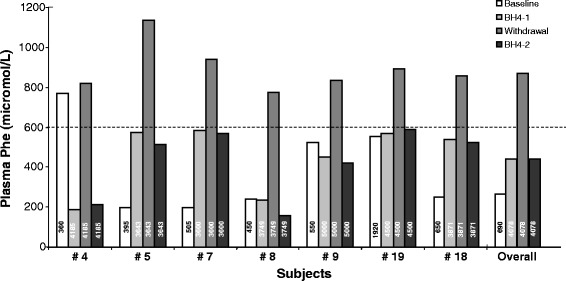
**BH4 withdrawal test in 7 patients.** Median Phe before BH4 therapy was calculated on Phe values collected during the 5 years prior to the enrolment into the trial (Baseline). Values reported in BH4-1 and Withdrawal represent the mean values of the Phe concentration at -10 and -5, and at +5 and +10 days from BH4 withdrawal, respectively. Patients were finally asked to re-start BH4 and to control plasma Phe levels 10 days later (day +20) (BH4-2). Numbers reported in the bars represent the daily Phe intake (mg/day) before BH4 therapy (white column) and the dietary Phe intake during the withdrawal test (dark columns). The dashed line indicates the plasma Phe upper value of the target range for the age group (600 micromol/L).

A BH4 withdrawal test was performed to add an additional proof of efficacy of sapropterin therapy. Before therapy withdrawal, all patients were in good metabolic control. During the test, all patients, under a constant diet, showed an increase of plasma Phe ranging from 31 to 87%. After the reintroduction of sapropterin, plasma Phe levels returned to pre-test values (Figure 2).

### Safety and tolerability

Both tetrahydrobiopterin (Schircks Laboratories) and sapropterin (Kuvan®, Merck-Serono) were generally safe and well tolerated. During the long-term follow-up, adverse events were recorded in 7 patients (41%). In three patients, symptoms were likely associated with the treatment. The first patient (#3), treated with sapropterin at 15 mg/kg/day, after 26 months of follow-up, complained of epigastric pain and nausea over a period of two months. A trial with ranitidine failed to reduce symptoms. Sapropterin dose was reduced to 10 mg/kg/day with clinical improvement and then withdrawn with complete resolution of symptoms. After 2 months of wellbeing, sapropterin therapy was reintroduced with no clinical problems. Two patients (#6, 13) complained of headache, a known side effect of sapropterin. Gastrointestinal symptoms also occurred in two male patients (#5, 17). Patients were admitted to the hospital for acute abdominal pain, increased neutrophil count and C-reactive protein, and ultrasonographic signs of acute appendicitis. Both patients temporary stopped BH4 therapy and recovered after antibiotic therapy with no need of surgery. After one month, they restarted therapy with no additional side effects. Adverse events, unlikely linked to the therapy, were reported in additional three patients. A first patient (#18), a 23-year-old boy treated for 5 years, developed mild anorexia and food avoidance. He never required hospitalization and his nutritional status remained good all over the period. Dosage was adjusted to the new weight and the drug was continued. A second male patient (#12) experienced psychotic symptoms after a four years follow-up. The symptoms recovered after pharmacological treatment and sapropterin was continued in order to keep Phe levels low. Finally, a third male patient (#7) was diagnosed with testicular seminoma.

Pregnancy was detected in a 23-year-old girl in follow-up for 24 months. Sapropterin was withdrawn at the 7^th^ week of gestational age. The patient kept a good metabolic control with diet alone throughout the pregnancy. The baby presented normal at birth and he is still in good health after a 12 months follow-up.

### Long term follow-up of BH4 non-responder patients

BH4 non-responder patients returned to the previous dietary regimen with the exception of the 6 subjects (# 22, 23, 25, 39, 40, 42) reported in Tables 1 and 2, who were selected for a longitudinal analysis of blood Phe at the new established Phe intake. Of these, one patient (#39) was excluded because he decided to start large neutral amino acids (LNAA) treatment. Data on the remaining 5 subjects are reported in Additional file 4. The increase of dietary Phe over a five years period ranged from 1.3 to 3-folds compared to the historical baseline. At the end of the follow-up, the Phe daily intake was in all cases >600 mg Phe/day. The metabolic control improved in two subjects (# 22, 25), remained stable in subjects #23 and 42 and worsened in subject #40, although the median plasma Phe was <400 micromol/L. Median blood Phe was <360 micromol/L in three subjects and between 360 and 380 micromol/L in the other two patients.

## Discussion

### Reassessment of Phe tolerance

Phe tolerance is not a clear-cut parameter, since it depends on the Phe target concentration that varies among countries and age-groups [27]. Blood Phe (and consequently Phe tolerance) may be influenced by protein catabolism in the neonatal period [30], illnesses, rapid growth during the first months of life and adolescence, and changes in body composition in adulthood. In the daily clinical practice, an unchanged Phe restricted diet may be prescribed for years if blood Phe concentrations remain within the target range, even though protein requirement may have increased [31]. Variation of Phe tolerance with increasing age is still an unsolved question. van Spronsen and colleagues in 2009 reported that Phe tolerance at 2, 3 and 5 years correlates with tolerance at 10 years [32]. To the best of our knowledge, Phe tolerance beyond adolescence was reassessed in only 8 adult PKU subjects [33]. Tolerance expressed as mg Phe/day increased in all cases with the exception of one patient. The authors speculated that dietary Phe intake was probably not adequate to support protein turnover for patients’ current body mass.

In our study, the BH4 loading test gave us the opportunity to question and re-evaluate our classification of some patients based on Phe tolerance, as defined at 5 years of age. First of all, re-evaluation of Phe tolerance was necessary only in a proportion of patients, namely in 6/24 BH4 non-responder patients and in 14/19 responders. With the exception of a 7 year-old male, patients’ age ranged from 12 to 24 years (Additional file 1). In the remaining patients, no variations of Phe tolerance were noted. Table 1 shows patients that under the increased dietary regimen maintained Phe values < 360 micromol/L, the same upper reference limit considered at 5 years of age and until adolescence. Although we recognize that two blood determinations are insufficient to draw definite conclusions, we can speculate that in this set of patients, phenotypic classification based on tolerance at 5 years of age was wrong and that those patients were unnecessary overtreated. Table 2 shows patients that under the new Phe intake showed blood Phe values between 360 and 600 micromol/L. Despite Phe levels were above the upper reference limit used at 5 years of age (when tolerance was assessed), Phe values remained within the target range for age-group.

Over a five years follow-up, 4 out of 5 patients improved their metabolic control or remained stable probably because of better adherence to diet, less catabolic processes or both. Finally, as recent data are drawing an intriguing scenario of interactions between PAH mutated enzyme and the substrate [34] a cooperative interaction between mutated alleles and Phe may not be excluded.

### BH4 loading test

#### Subjects

Responsiveness to BH4 was observed in all phenotypic classes, including 9.5% of cPKU subjects. This observation retraces previous reports of BH4 response also in cPKU [18,23]. In addition, more than half of the enrolled patients with moPKU responded to BH4, showing that the phenotype alone is not a good predictor of response to BH4, but other factors must be taken into consideration.

#### Genotypes

The presence of at least one mutation with residual enzymatic activity resulted the best predictor of BH4-responsiveness in our cohort of patients. Residual PAH activity was strongly associated with response, while the presence of two inactive alleles excluded responsiveness. As expected, missense mutations were prevalent among BH4 responder patients (95%), while splice mutations were prevalent among non-responders. The p.R261Q mutation combined with a missense mutation was always associated with responsiveness while, when associated with a splice mutation, it was associated with non-responsiveness, except for the case of patient #11, who resulted a pseudo-responder (Additional file 1). The p.L48S mutation was found in BH4 responder patients except for one non-responder patient, homozygous for this PAH variant. The p.Y414C mutation was associated with BH4 responsiveness in the START study [35], but non-responder patients carrying the p.Y414C mutation in heterozygosity were also described [36]. In our study, the same genotype (p.R158Q/p.Y414C) was found both in a responder and in a non-responder patient (#16, 34). In this case the possible action of modifier genes cannot be excluded.

#### Rate of response

The use of a loading test extended to 48 h was important to detect responsiveness in both the patients with severe PKU phenotypes and those with mPKU/mHPA. Indeed, 4 mPKU and 1 mHPA patients would have been missed if a 24 h test had been performed. Only one pseudo-responder patient (#11) was identified (2%). Patients who cannot increase their tolerance despite a significant decrease of plasma Phe during the BH4 loading test are emerging in the clinical landscape. So far, 3 out of 14 patients (21%), responders to a 24 h loading test [37], 2 out of 16 (12.5%) patients, responders to an 8-day BH4 load [14], and 9 out of 58 patients (15.5%), responders to a 1 month BH4 load [17] were unable to increase their tolerance on long-term therapy. The low incidence of pseudo-responders found in the present study suggests that a 48 h-long loading test performed in a hospital setting may minimize the risk of false positive responses.

#### Predictors of responsiveness

The ability to predict BH4 response in PKU patients could reduce costs and help guide management of PKU patients. Besides the impact of the genotype, our results show that the biochemical phenotype and lower Phe/Tyr ratio at T0 of the test were significantly different in BH4 responders compared to non-responders.

### Long-term therapy with BH4

So far, published papers showed a positive effect of sapropterin in reducing Phe levels in BH4-responders. Poor data are available on long-term clinical outcomes [1]. Few studies described the long-term effect of sapropterin therapy on tolerance [14,15,38].

The present study showed increased natural protein intake over the long-term period. Phe tolerance increased also in patients with severe forms of HPA. Despite the dramatic increase of dietary Phe, in 11 patients (59%) plasma Phe values decreased or remained stable (Table 5). In the remaining patients (#2, 6, 7, 10, 11, 12), plasma Phe increased but remained below the Phe target for age considered in this study. In patient #7, results of the BH4 withdrawal test demonstrated the efficacy of BH4 therapy; patient #11 was later shown to be a pseudo-responder. However, in patients #2, 6, 10 and 12, we failed to prove definitively the beneficial effect of BH4 therapy although, under BH4, a good metabolic control was generally maintained. Adolescence and adulthood are critical periods for PKU management due to poor compliance to diet and inadequate metabolic control. Diet relaxation and optimal metabolic control under sapropterin therapy is particularly beneficial in these groups of patients and may improve quality of life and neuropsychological performances [39].

Eighty-eight percent of patients had a better metabolic control if the daily BH4 dosage was divided in three doses. It was suggested that the curve of Phe reduction during the BH4 loading test could predict if 1, 2 or 3 doses/day of BH4 had to be prescribed [28]. However, in our study, all BH4-responder patients showed a gradual and progressive reduction of Phe values during the test with no evidence of peaks that could let us discriminate *a priori* the need of a single *vs* triple BH4 dose per day.

Patients who resulted as BH4 responders and started the long-term treatment during adolescence maintained the same metabolic control at the same dietary regimen during adulthood. This observation suggests that a higher growth rate does not explain the observed increase of tolerance to Phe.

### BH4 withdrawal test

The improvement of dietary Phe intake and of metabolic control was directly dependent on BH4 assumption, as further demonstrated by the BH4 withdrawal test. Under a constant dietary regimen, the BH4 withdrawal test, performed for the first time in the present study, clearly showed an increase of plasma Phe ranging from 31 to 87%. At the resumption of therapy, Phe values readily returned to the previous plasma levels.

### Safety and tolerability

The Kuvan® safety sheet reports headache, rhinorrhea, pharyngolaryngeal pain, gastrointestinal symptoms, cough and nasal congestion as the most common adverse reactions to sapropterin. In this study, gastrointestinal symptoms (epigastric pain and appendicitis-like symptoms) were reported in three patients (17.6%). In the study by Burton et al. [9], appendicitis was first described in one patient 1 month after the discontinuation of the drug. However, appendicitis is a common disease in the general population and additional follow-up of large cohorts of patients is required to ascertain whether the symptoms are sapropterin-related or not. Headache was found in nearly 12% of patients. Other adverse events were transient anorexia and psychotic symptoms. Psychiatric symptoms have been reported across the lifespan of individuals with PKU, especially during adulthood, even among the early treated ones [40] and probably the observed adverse effects are not linked to sapropterin. In one patient, symptoms (epigastralgia and nausea) regressed after drug discontinuation and did not re-occurred after the resumption of the treatment showing that, in case of discontinuation of the treatment due to a drug- related side effect, the attempt to reintroduce sapropterin may be done.

Maternal PKU syndrome is still an issue in PKU management, since a strict metabolic control should be maintained throughout pregnancy. Sapropterin has been proposed as an adjuvant to diet for those women who are unable to achieve low Phe levels, but safety data for the foetus are scarce. A recent study reported 8 cases of PKU woman treated with sapropterin throughout pregnancy who gave birth to normal children in 7 cases and to a child affected with Potter syndrome in one case [41]. Here we describe a case of a women treated with Kuvan® until the 7^th^ week of gestational age, a critical time window for embryonic development, who gave birth to a healthy child.

## Conclusions

In conclusion, results from this clinical trial of long-term treatment with BH4 confirmed that BH4 is safe and effective in increasing tolerance to Phe while keeping a good metabolic control. The improvement of Phe tolerance is due to BH4 assumption, as shown by the BH4 withdrawal test.

As an incidental observation, we reassessed Phe tolerance in a subset of PKU patients. Accurate phenotyping of PKU patients is difficult and insidious and no consensus exists among different Metabolic Centers. However, there is increasing need to assess the real tolerance to Phe in PKU patients to ameliorate quality of life, improve nutritional status, avoid unnecessarily restricted diets, and interpret the effect of new therapies for PKU.

## Additional files

Additional file 1:
**Phenotypic and genotypic characterization of HPA subjects.**
Additional file 2:
**Study diagram.**
Additional file 3:
**Body mass index (BMI) of patients on long-term treatment with BH4.**
Additional file 4:
**Tolerance and plasma Phe values before and after re-evaluation of Phe tolerance in five patients non-responders to BH4: a five years follow-up.** Columns represent dietary Phe intake expressed in mg/day. Lines represent median Phe values (micromol/L) and 10th-90th centile; medians were calculated from plasma Phe values collected during the 5 years prior to the progressive increase of dietary Phe and during the 5 years follow-up after the achievement of the new dietary intake, respectively. All median values under the new dietary regimen fell into the reference range of plasma Phe for age group.

## Abbreviations

PKU: Phenylketonuria
PAH: Phenylalanine hydroxylase
HPA: Hyperphenylalaninemia
BH4: Tetrahydrobiopterin
Phe: Phenylalanine
Tyr: Tyrosine
