# Supplementary material for: Long-term follow-up of patients with phenylketonuria treated with tetrahydrobiopterin: a seven years experience
Source: Orphanet J Rare Dis. 2015 Feb 8;10:14. doi: 10.1186/s13023-015-0227-8 (PMC4351928; doi:10.1186/s13023-015-0227-8)
Supplement: Additional file 1: — Phenotypic and genotypic characterization of HPA subjects. [file 13023_2015_227_MOESM1_ESM.pdf]

Additional file 1. Phenotypic and genotypic characterization of HPA subjects

Subject (#)	Gender	Phenotype	Plasma Phe at diagnosis (micromol/L)	Tolerance (mg Phe/day)	Allele 1	PRA (%)	Allele 2	PRA (%)	Age at loading test (years)
1	F	cPKU	1260	280	R261Q	44	P281L	2	13
2	M	cPKU	1860	325	R261Q	44	P281L	2	11
3	F	moPKU	1176	400	R261Q	44	R158Q	10	17
4	F	moPKU	1190	360	R261Q	44	L48S	39	14
5	M	moPKU	1080	395	R261Q	44	P281L	2	14
6	M	moPKU	2100	385	L48S	39	Q301P	4,4	7
7	M	mPKU	650	505	L48S	39	R158Q	10	14
8	F	mPKU	700	450	L48S	39	D222G	n.d.	12
9	M	mPKU	696	550	165delT	n.d.	P366H	n.d.	18
10	M	mPKU	1158	410	R261Q	44	R261Q	44	13
11	F	mPKU	1860	440	R261Q	44	IVS10nt-11G>A	0	20
12	M	mPKU	1200	410	R261Q	44	P281L	2	17
13	F	mPKU	1800	410	L48S	39	R158Q	10	21
14	M	mPKU	1320	440	L48S	39	R158Q	10	10
15	F	mPKU	450	400	L48S	39	L48S	39	5
16	F	mPKU	720	410	R158Q	10	Y414C	57	12
17	M	mHPA	590	1500	R158Q	10	D338Y	n.d.	14
18	M	mHPA	550	650	L48S	39	R158Q	10	19
19	M	mHPA	380	1920	165delT	n.d.	P366H	n.d.	22
20	F	cPKU	2184	345	R261X	0	IVS10nt-11G>A	0	17
21	F	cPKU	2130	340	R261Q	44	IVS07nt3G>C	0	17
22	F	cPKU	1500	345	R261Q	44	IVS10nt-11G>A	0	16
23	M	cPKU	1680	340	R261Q	44	IVS10nt-11G>A	0	12
24	F	cPKU	1500	280	R252W	0	R408W	<1	13
25	M	cPKU	2850	320	R158Q	10	R176X	0	16
26	F	cPKU	1662	230	IVS10nt-11G>A	0	IVS10nt-11G>A	0	13
27	M	cPKU	2160	320	IVS10nt-11G>A	0	R261X	0	16
28	F	cPKU	1920	330	IVS10nt-11G>A	0	L213P	n.d.	18
29	F	cPKU	1800	265	R261Q	44	IVS10nt-11G>A	0	20
30	F	cPKU	1200	285	P281L	2	P281L	2	16
31	M	cPKU	1200	291	ISV10nt546	<1	R261Q	44	15
32	M	cPKU	1560	324	pS16>xFsx1	<1	IVS7+3G>C	n.d.	14
33	F	cPKU	4272	220	R158Q	10	R158Q	10	11
34	M	cPKU	1200	258	R158Q	10	Y414C	57	5
35	F	cPKU	900	291	ISV10nt546	<1	ISV10nt546	<1	16
36	F	cPKU	1200	220	ISV10nt546	<1	ISV10nt546	<1	5

37	F	cPKU	1180	223	ISV10nt546	<1	ISV10nt546	<1	7
38	F	cPKU	1220	270	ISV10nt546	<1	165delT	<1	8
39	M	moPKU	1800	390	P281L	2	R158Q	10	14
40	M	moPKU	1200	390	F39del(116-118delTCT)	20	IVS10nt-11G>A	0	12
41	F	mPKU	1800	505	W187X	0	P281L	2	17
42	F	mHPA	1500	630	IVS06nt-2delA	n.d.	P281L	2	24
43	M	mHPA	420	620	L48S	39	L48S	39	7

Note: Patients were phenotypically classified according to plasma Phe level at diagnosis and Phe tolerance calculated at 5 years of age. When Phe at diagnosis was inconsistent with Phe tolerance, patients were classified according to the latter parameter. The predictive enzymatic activity (PRA) is the average of the data obtained in eukaryotic cells, as reported in the BIOPKU database (http://www.biopku.org) and in Daniele et al, 2006. n.d.: not determined. Patients # 1 to 19 resulted as BH4 responders; patients # 20 to 43 resulted as BH4 non-responders.
