# Supplementary figures and images for: Long-term follow-up of patients with phenylketonuria treated with tetrahydrobiopterin: a seven years experience
Source: Orphanet J Rare Dis. 2015 Feb 8;10:14. doi: 10.1186/s13023-015-0227-8 (PMC4351928; doi:10.1186/s13023-015-0227-8)

**Additional file 2. Study diagram**

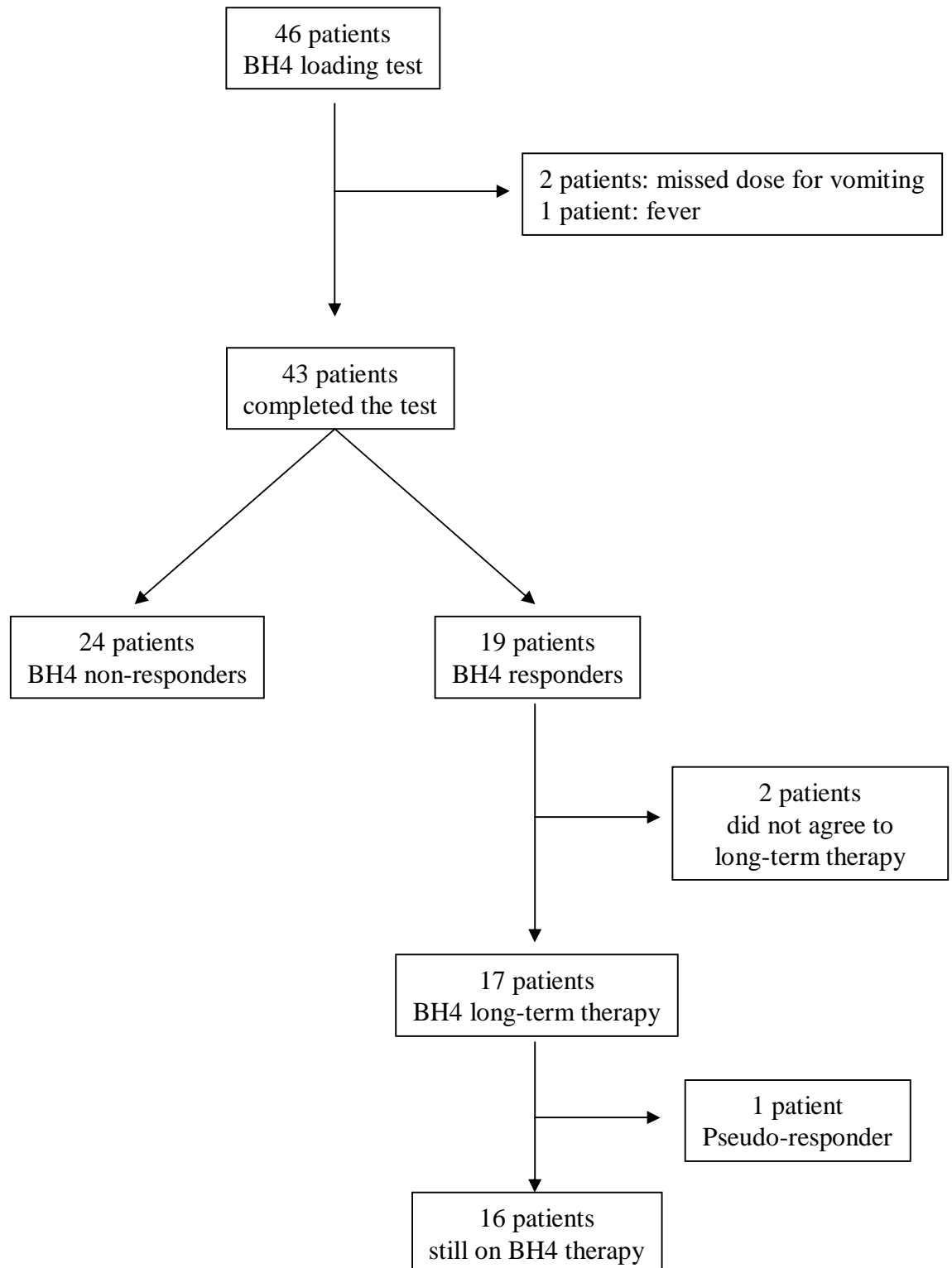

Supplement: Additional file 2: — Study diagram. [file 13023_2015_227_MOESM2_ESM.pdf]

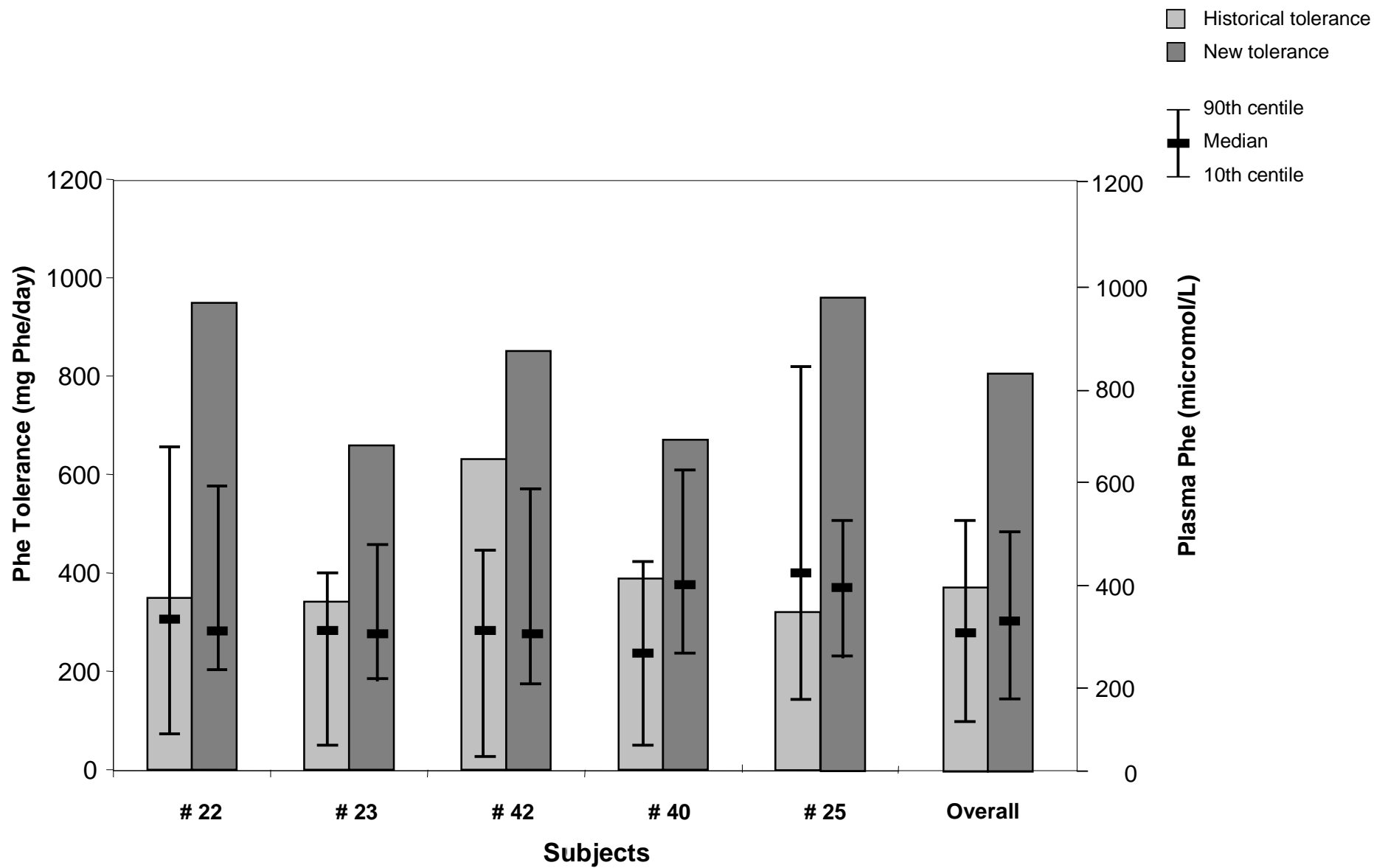

Supplement: Additional file 4: — Tolerance and plasma Phe values before and after re-evaluation of Phe tolerance in five patients non-responders to BH4: a five years follow-up. Columns represent dietary Phe intake expressed in mg/day. Lines represent median Phe values (micromol/L) and 10th-90th centile; medians were calculated from plasma Phe values collected during the 5 years prior to the progressive increase of dietary Phe and during the 5 years follow-up after the achievement of the new dietary intake, respectively. All median values under the new dietary regimen fell into the reference range of plasma Phe for age group. [file 13023_2015_227_MOESM4_ESM.pdf]
