# Supplementary material for: Long-term follow-up of patients with phenylketonuria treated with tetrahydrobiopterin: a seven years experience
Source: Orphanet J Rare Dis. 2015 Feb 8;10:14. doi: 10.1186/s13023-015-0227-8 (PMC4351928; doi:10.1186/s13023-015-0227-8)
Supplement: Additional file 3: — Body mass index (BMI) of patients on long-term treatment with BH4. [file 13023_2015_227_MOESM3_ESM.pdf]

Additional file 3. Body mass index (BMI) of patients on long-term treatment with BH4

	**BMI (**mean±SD) **Pre –BH4***	**BMI (**mean±SD) **On BH4**	**p-value**
**Overall patients**	21.7±2.1	23.6±1.39	**0.006**
cPKU	23.29±0.19	24±0.49	0.15
moPKU	20.5±1.5	23.4±1.2	**0.02**
mPKU	21±1.3	23±1.5	**0.008**
HPA	24.8±2.5	24.6±1.5	0.9
**Patients on BH4+DIET**	21±1.7	23.5±1.5	**0.01**
**Patients on BH4 alone**	22.5±2.5	23.8±1.3	0.2

* 5 years before start of treatment
